# Renal denervation as a treatment strategy for vasospastic angina induced ventricular tachycardia

**DOI:** 10.1007/s12471-017-1012-1

**Published:** 2017-06-12

**Authors:** L. Feyz, S. Wijchers, J. Daemen

**Affiliations:** Department of Cardiology, Thorax Center, Erasmus Medical Center, Rotterdam, The Netherlands

**Fig. 1:**
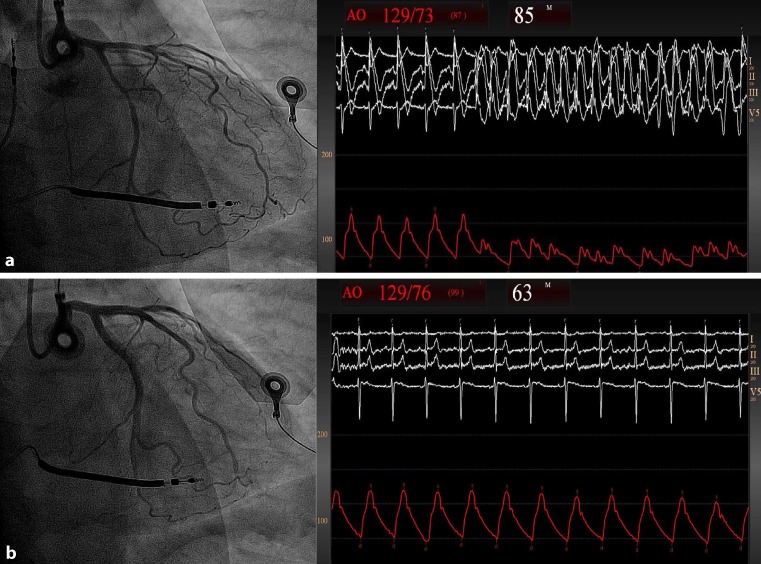
Methylergometrine testing revealed severe coronary spasms in multiple coronary segments resulting in ventricular tachycardia and haemodynamic collapse (**a**). This quickly resolved after intracoronary nitrates (**b**)

Polymorphic ventricular tachycardia can be a detrimental consequence of coronary vasospasm [1]. The pathophysiology of vasospastic angina (VA) is poorly understood, although the disease has been linked to sympathetic hyperactivity [2–4]. We present a 52-year-old male smoker with ventricular fibrillation (VF) 2 years ago. Work-up revealed no signs of structural heart disease or obstructive coronary disease. We implanted an implantable cardioverter defibrillator (ICD). Several episodes of non-sustained polymorphic ventricular tachycardia and ICD shocks due to VF ensued, despite maintenance therapy with isosorbide 100 mg, metoprolol 50 mg, verapamil 300 mg, amiodarone 200 mg. Methylergometrine testing confirmed VA as the cause of VF (Fig. 1a,b). To reduce sympathetic hyperactivity, bilateral renal denervation was performed using the ReCor Paradise system. The patient remained free from episodes of angina and ventricular arrhythmias at 6 and 12 months. Ambulatory blood pressure and mean heart rate remained stable between baseline and 6 months (108/60 mm Hg vs. 113/71 mm Hg and 60 bpm vs. 60 bpm, respectively). Renal denervation could be a safe and effective treatment for normotensive patients with severe VA despite optimal medical therapy.
